# AMH Concentrations in Peritoneal Fluids of Women With and Without Endometriosis

**DOI:** 10.3389/fsurg.2020.600202

**Published:** 2020-11-11

**Authors:** Michio Kitajima, Kanako Matsumoto, Naoko Murakami, Itsuki Kajimura, Ayumi Harada, Yuriko Kitajima, Hideaki Masuzaki, Kiyonori Miura

**Affiliations:** ^1^Department of Obstetrics and Gynecology, Nagasaki University Graduate School of Biomedical Sciences, Nagasaki, Japan; ^2^Department of Obstetrics and Gynecology, Nagasaki University Hospital, Nagasaki, Japan

**Keywords:** AMH (anti-Müllerian hormone), peritoneal fluid, endometriosis, AMH receptor type2 (AMHR2), peritoneal lesions

## Abstract

**Background:** As its name indicates, anti-Müllerian hormone (AMH) is primarily found as an inhibitor of the Müllerian duct in male fetus. On the other hand, AMH may act as a mediator of Müllerian duct-derived female tissue, such as endometrium in normal and pathological conditions. However, the role of AMH in the functional regulations of endometriosis is not well understood. It can be hypothesized that AMH in peritoneal fluids may affect the activity of peritoneal endometriosis. In this study, we investigated the levels of AMH in peritoneal fluids (PF) in women with and without endometriosis.

**Methods:** PF were collected during laparoscopy from 90 women diagnosed as having advanced endometriosis (rASRM stage III, *n* = 30; stage IV, *n* = 60), and 32 women without endometriosis were served as control. Paired serum samples were also collected before the surgery. AMH in PF and serum were measured by ELISA. Individual clinical information was collected. AMH levels were compared according to the presence of endometriosis. The expression of AMHR2 in peritoneal endometriotic lesions obtained during laparoscopy was examined by immunohistochemistry.

**Results:** AMH levels in PF were positively and significantly correlated with serum AMH levels in both women with and without endometriosis (*R*^2^ = 0.17, *P* < 0.0001; *R*^2^ = 0.30, *P* = 0.001, respectively). Serum AMH levels were inversely and significantly correlated with age in women with endometriosis (*R*^2^ = 0.092, *P* = 0.004) and in control women without statistical significance (*R*^2^ = 0.078, *P* = 0.12). AMH levels in PF were also inversely but not significantly correlated with age in women with and without endometriosis (*R*^2^ = 0.029, *P* = 0.11 and *R*^2^ = 0.027, *P* = 0.37, respectively). Mean age and serum AMH levels were not significantly different between two groups. On the other hand, AMH levels in PF were significantly lower in women with endometriosis compared to those of control women [2.15 ± 2.13 (mean ± SD) vs. 4.40 ± 4.77 ng/mL, *P* = 0.0001]. AMHR2 are localized at glandular epithelium and stromal cells in the ectopic endometrium of peritoneal endometriosis.

**Conclusions:** Women with endometriosis may present lower PF AMH levels even if they retain serum levels similar to women without disease. As peritoneal endometriosis expresses a specific receptor for AMH, lower AMH levels in PF of women with advanced endometriosis may be involved in the pathophysiology of peritoneal endometriosis.

## Introduction

Endometriosis is an estrogen-dependent chronic inflammatory disease. It may affect 5–10% of women of reproductive age (1). The disease is manifested by varieties of pain symptom, such as dysmenorrhea, dyschesia, dyspareunia, and chronic pelvic pain. It is also found in up to 50% of women with infertility (2). Retrograde menstrual flow via fallopian tubes is believed to play main roles in the development of peritoneal endometriosis, though the pathogenesis of the disease remains controversial.

Anti-Müllerian hormone (AMH) is a dimeric glycoprotein which belongs to the transforming growth factor-β superfamily. AMH was primarily found as a substance involved in the regression of the Müllerian duct in the male fetus produced from Sertoli cells (3). In female, the granulosa cells of early growing ovarian follicles are the sole source of serum AMH levels, which declines with age. Serum AMH levels are utilized as a marker of ovarian reserve in clinical infertility care (4).

On the other hand, AMH may act as a functional mediator of female reproductive tissue derived from the Müllerian duct, such as the ovarian epithelium, pelvic peritoneum, and endometrium. There have been several reports describing the potential inhibitory role of AMH on normal and pathological conditions of the uterus and ovary including cancer (5, 6). It may exert its effect via a specific receptor designated as AMH receptor (AMHR) type I and II, where the type II receptor (AMHR2) exerts its function in specific binding to AMH (7). The expression of AMHR2 in human tissue including Müllerian duct-derived female reproductive organs, such as endometrium, had been reported (8). However, the role of AMH in the functional regulations of human endometrium is not well understood.

Peritoneal fluids (PF) in women with endometriosis may contain a series of inflammatory cytokines and chemokines, which are involved in the growth and progression of endometriotic lesions (9). These macromolecules are derived from ectopic endometrial glandular cells, stromal cells, surrounding vascular cells, and infiltrated macrophages. At the same time, inhibitors against these stimulants may also be produced. Peritoneal fluids may work as a reservoir of growth mediators of endometriotic lesions. Thus, the balance between stimulants and inhibitors in PF may affect the growth abilities of endometriotic lesions.

Therefore, it can be hypothesized that different levels of AMH in PF may affect the growth and progression of peritoneal endometriosis. In this study, we investigated the levels of AMH in PF in women with and without endometriosis and compared them according to the age of the subjects and the presence of disease. We also studied the expression of a specific receptor of AMH (AMH receptor type 2, AMHR2) in peritoneal endometriosis. The aim of this study is to delineate the possible involvement of AMH in the pathogenesis of peritoneal endometriosis.

## Materials and Methods

We performed a retrospective analysis using archivally stored paired samples of serum and PF from women diagnosed as having advanced endometriosis and women without endometriosis, which were collected during laparoscopic surgery from 2012 to 2018. Written consent for the usage of these samples for the research purpose was obtained, and the institutional review board approved this study. In women with endometriosis, the severity of the disease was classified according to the rASRM scoring system at the time of laparoscopy. Endometriotic lesions were excised and coagulated by bipolar energy devices. Control women had laparoscopy for benign gynecological conditions, such as benign ovarian tumor, uterine fibroids, uterine anomaly, and unexplained infertility.

PF were collected at the beginning of the surgery and centrifuged at 3,000 g for 10 min, then the supernatant was stored at −80°C until the examinations. AMH in PF were measured by ELISA (AMH Gen II ELISA, Beckman Coulter). Blood samples were collected before the surgery and were centrifuged and sera were stored at −80°C, and serum AMH levels were measured in a similar way to PF samples. Individual clinical backgrounds, operative findings, and pathological diagnosis after the surgeries were collected from patients' records. To eliminate the effects of subjects with abnormally higher AMH production, women with solid ovarian tumor or irregular menstruation with serum AMH levels higher than 10.0 ng/mL were excluded from the present study. The differences between AMH in PF and serum AMH in individual subjects were calculated by subtraction (AMH in PF minus serum AMH).

Peritoneal endometriotic lesions were obtained during laparoscopy. The tissues were fixed in 10% formalin and embedded in paraffin, and 5-μm thick sections were made. Hematoxylin and eosin (H.E.) staining sections were made for routine pathological diagnosis. The tissue samples with intact endometrial glandular epithelial cells and stromal cells were selected for the examination of the expression of AMHR2 by immunohistochemistry as previously described (10). Briefly, 5-μm sections were pretreated in 0.05% citraconic anhydride solution (Immunosaver, Nissin EM) for antigen retrieval. After blocking endogenous peroxidase activity and nonspecific binding, they were incubated with rabbit polyclonal anti-AMHR2 antibody [AMHR2 (N-term), Rabbit poly ABG-AP7111a, Life Span Biosciences] overnight. After washing the sections, they were incubated with peroxidase-labeled polymer conjugated to goat anti-rabbit immunoglobulins (EnVision; DAKO) and washed as previously described. Specific immunoreactivity was visualized by 3,3′-diaminobenzidine tetrahydrochloride. The sections were counterstained with Mayer's hematoxylin. For negative controls, sections were incubated with rabbit IgG instead of specific antibodies at the same dilution. Positive controls consisted of a full-thickness human endometrium derived from a hysterectomized uterus were used.

For statistical analysis, continuous variables are compared with unpaired student-*t* test and Mann–Whitney U test. Categorical variables are compared with kai square test and Fisher's exact test. Linear regression analyses were performed to detect a significant correlation between two continuous variables. All statistical analyses were performed with computer software (JMP Pro 14.0.0, SAS institute Japan, Tokyo). *P* values under 0.05 were considered as statistical significance.

## Results

Paired serum and PF AMH levels were examined in 90 women with advanced endometriosis (rASRM stage III, *n* = 30; stage IV, *n* = 60) and 32 control women. Indications of surgery in control women were benign ovarian tumor (*n* = 15), uterine fibroids (*n* = 9), and diagnostic laparoscopy for uterine anomaly or infertility (*n* = 8). The clinical backgrounds of study subjects are summarized in Table 1. We did not find a statistically significant difference in the distributions of age between two groups. The proportions of women with infertility were significantly higher in women with stage IV (*n* = 42, 70%) compared to those of women with stage III and control (*n* = 12, 40%, *p* = 0.006, and *n* = 14, 44%, *P* = 0.01, respectively). In women with endometriosis, various sizes of endometriomas were found in 84 women [26 (87%) in stage III and 58 (97%) in stage IV].

**Table 1:** Clinical backgrounds and AMH levels in serum and peritoneal fluids.

	**Endometriosis**		**Control**
Stage^*^ of endometriosis	III	IV	
Number of subjects	30	60	32
Mean age, years old (range)	32.4 ± 4.7		32.0 ± 6.9 (15–44)
	31.0 ± 4.4 (24–41)	33.1 ± 4.7 (21–45)	
Subjects >35 years old	7 (23%)	23 (38%)	12 (38%)
Subjects with infertility	12 (40%)	42 (70%)^†^	14 (44%)
Subjects with endometriomas	26 (87%)	58 (97%)	NA
Mean lesion score by rASRM system (range)	21.6 ± 6.5 (6–38)	33.4 ± 11.0 (6–58)	NA
Mean adhesion score by rASRM system (range)	8.3 ± 6.0 (0–28)	44.7 ± 26.5 (6–96)	NA
Mean serum AMH levels, ng/mL (range)	2.90 ± 2.21		3.17 ± 2.93 (0.02–9.82)
	3.57 ± 2.40 (0.19–9.79)	2.57 ± 2.05 (0.1–9.91)	
Mean PF AMH levels, ng/mL(range)	2.15 ± 2.13^‡^		4.40 ± 4.77^‡^ (0.01–18.2)
	2.11 ± 1.47 (0.01–5.56)	2.17 ± 2.41 (0.01–11.4)	

*Values are expressed as mean ± SD (standard deviation); NA, not applicable; PF, peritoneal fluids*.

* *Stage of endometriosis were classified according to the revised ASRM (American Society of Reproductive Medicine) scoring system*.

† *P = 0.006 vs. stage III, P = 0.01 vs. control, kai square test*.

‡ *P = 0.0002, student-t test*.

**Figure 1:**
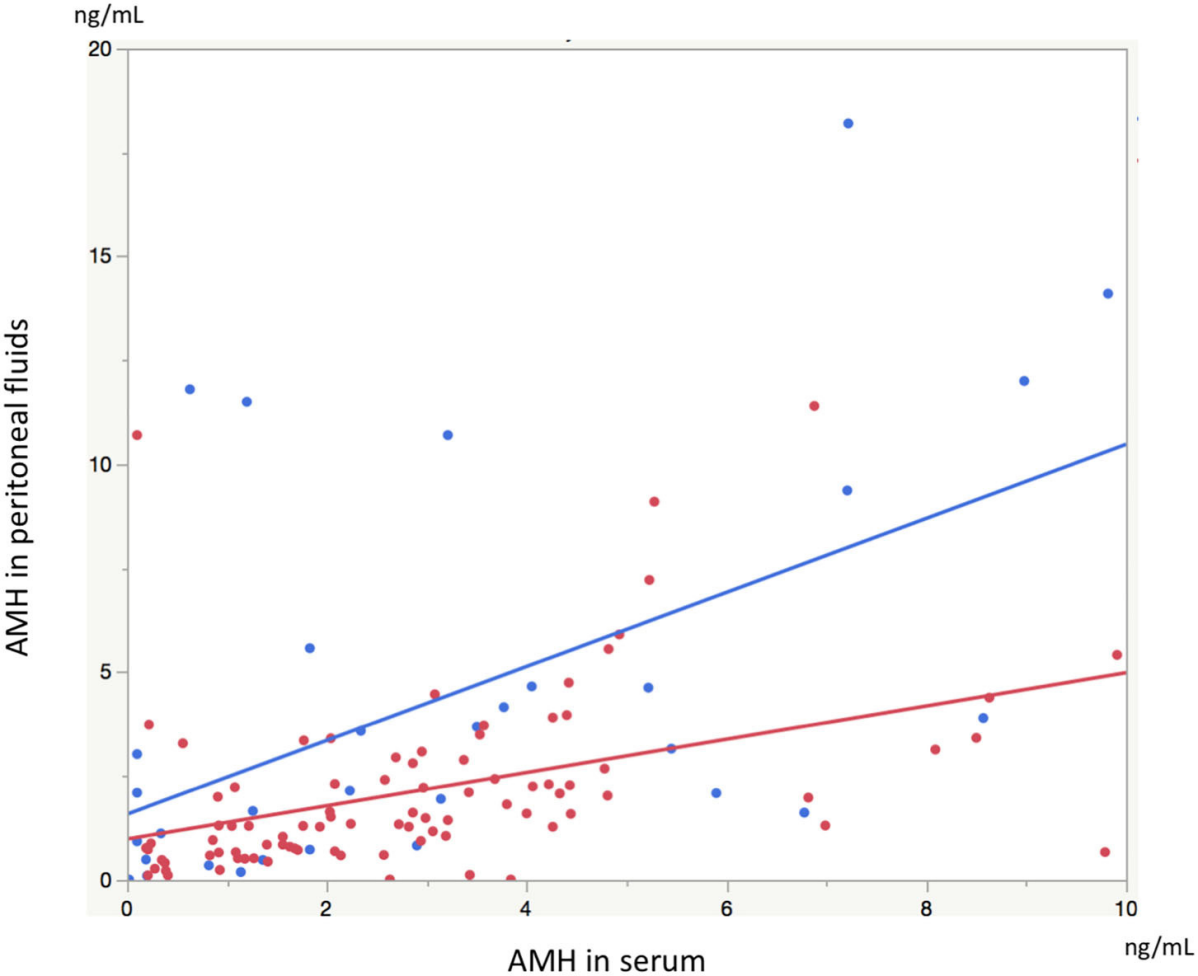
The graph shows linear correlation between serum AMH levels and AMH levels in peritoneal fluids (PF) in women with endometriosis (red dot) and without endometriosis (blue dot).

**Figure 2:**
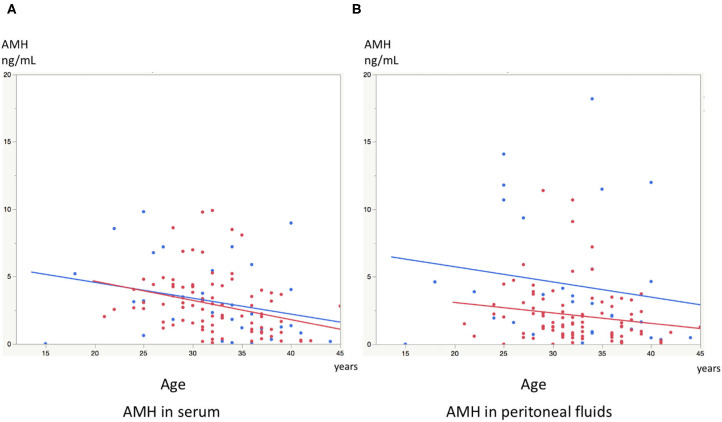
**(A)** The graph shows linear inverse correlation between serum AMH levels and age in women with endometriosis (red dot) and control women without disease (blue dot). **(B)** The graph shows linear inverse correlation between AMH levels in peritoneal fluids and age in women with endometriosis (red dot) and control women without disease (blue dot).

**Figure 3:**
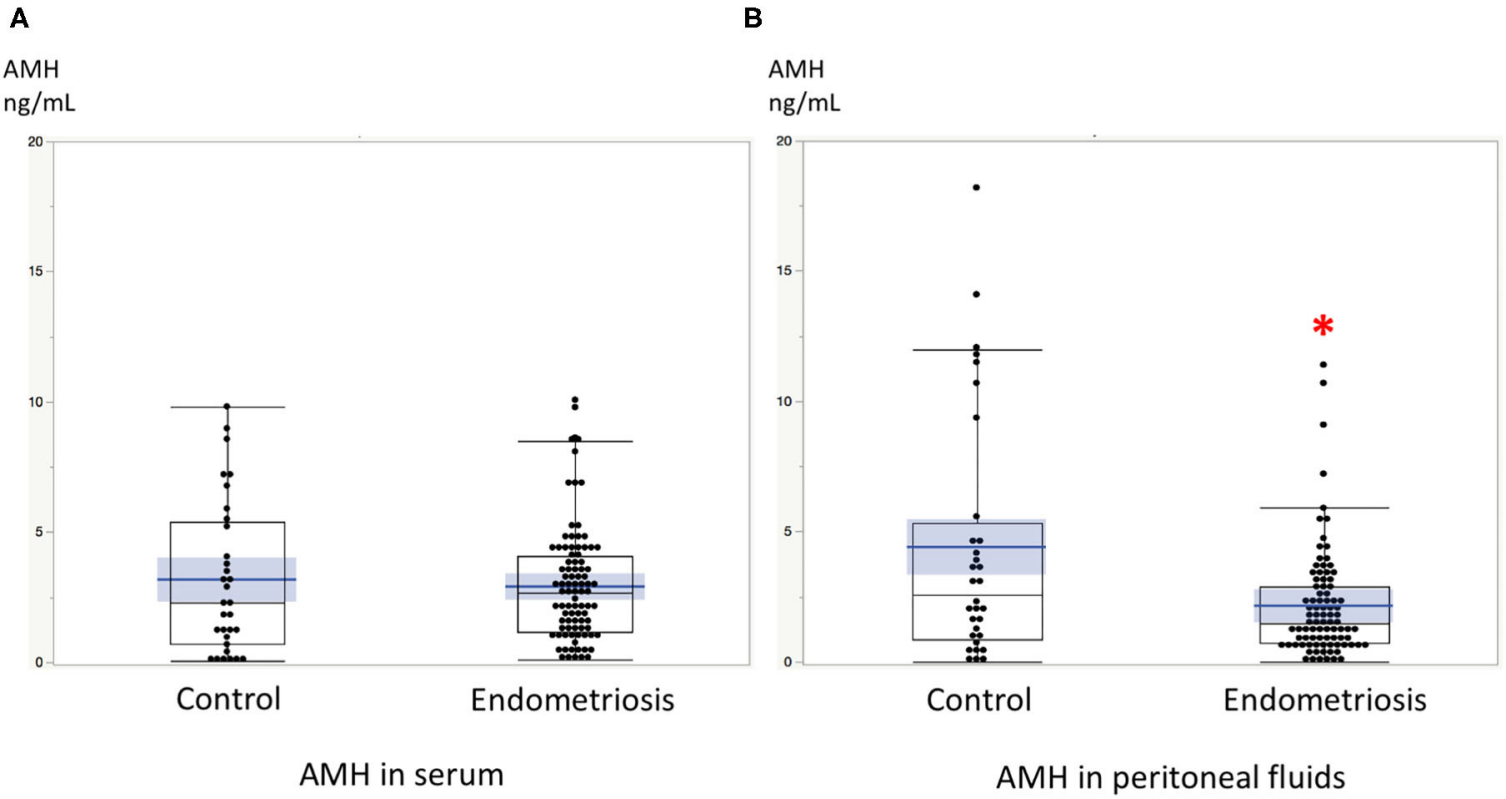
**(A)** The comparison of serum AMH levels between women with endometriosis and control women without disease. **(B)** The comparison of AMH levels in peritoneal fluids between women with endometriosis and control women without disease. *AMH levels in PF were significantly lower in women with endometriosis compared to those of control women without disease. Boxes represent the distance (interquartile range) between the first (25%) and third (75%) quartiles, and horizontal lines in the boxes represent median values. Blue horizontal line represents mean value and blue-colored square box represents 95% confidence interval. Each dot represents the exact value of individual cases.

**Figure 4:**
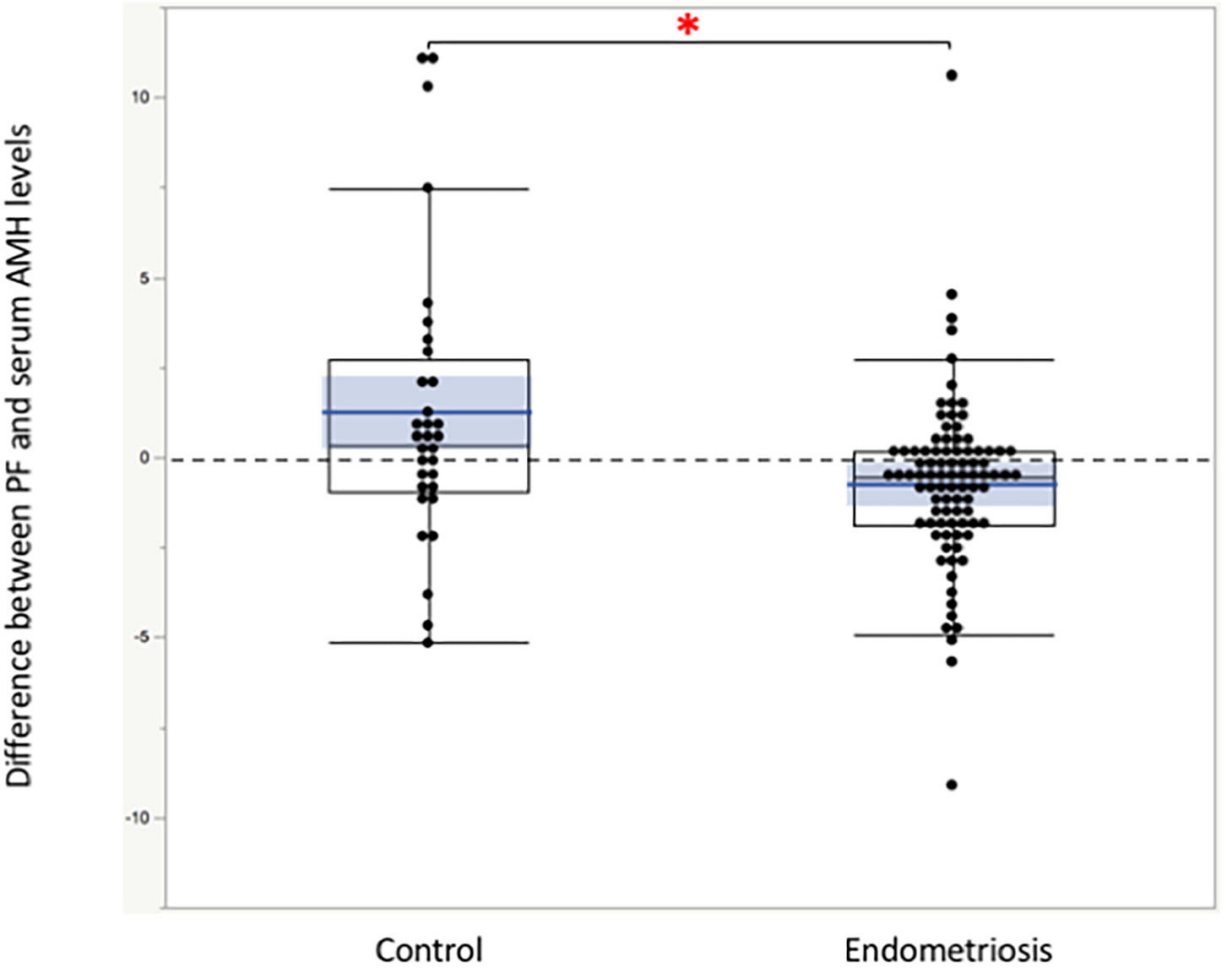
The comparison of the individual difference between AMH in peritoneal fluids and serum AMH calculated by subtraction. *The differences were significantly lower in women with endometriosis compared to those of control women without disease. Boxes represent the distance (interquartile range) between the first (25%) and third (75%) quartiles, and horizontal lines in the boxes represent median values. Blue horizontal line represents mean value, and blue-colored square box represents 95% confidence interval. Each dot represents exact value of individual case.

Significant linear correlations were found between the paired samples (serum AMH and AMH in PF) in women both with and without endometriosis (*R*^2^ = 0.17, *P* < 0.0001; *R*^2^ = 0.30, *P* = 0.001, respectively, Figure 1). Serum AMH levels were inversely and significantly correlated with age in women with endometriosis (*R*^2^ = 0.092, *P* = 0.004) and in control women without statistical significance (*R*^2^ = 0.078, *P* = 0.12). AMH levels in PF were also inversely but not significantly correlated with age in women with and without endometriosis (*R*^2^ = 0.029, *P* = 0.11 and *R*^2^ = 0.027, *P* = 0.37, respectively, Figure 2). We did not find a significant difference in serum AMH levels according to the presence of endometriosis [endometriosis 2.90 ± 2.21 (mean ± SD) ng/mL vs. control 3.17 ± 2.93 ng/mL, *P* = 0.29 (Figure 3A)]; however, AMH levels in PF were significantly lower in women with endometriosis compared to those of control women [endometriosis 2.08 ± 2.06 (mean ± SD) vs. control 4.40 ± 4.77 ng/mL, *P* = 0.0002 (Figure 3B)]. Individual differences between AMH in PF and serum AMH were significantly lower in women with endometriosis [endometriosis −0.75 ± 2.35 (mean ± SD) vs. control 1.23 ± 4.01, *P* = 0.001 (Figure 4)].

**Figure 5:**
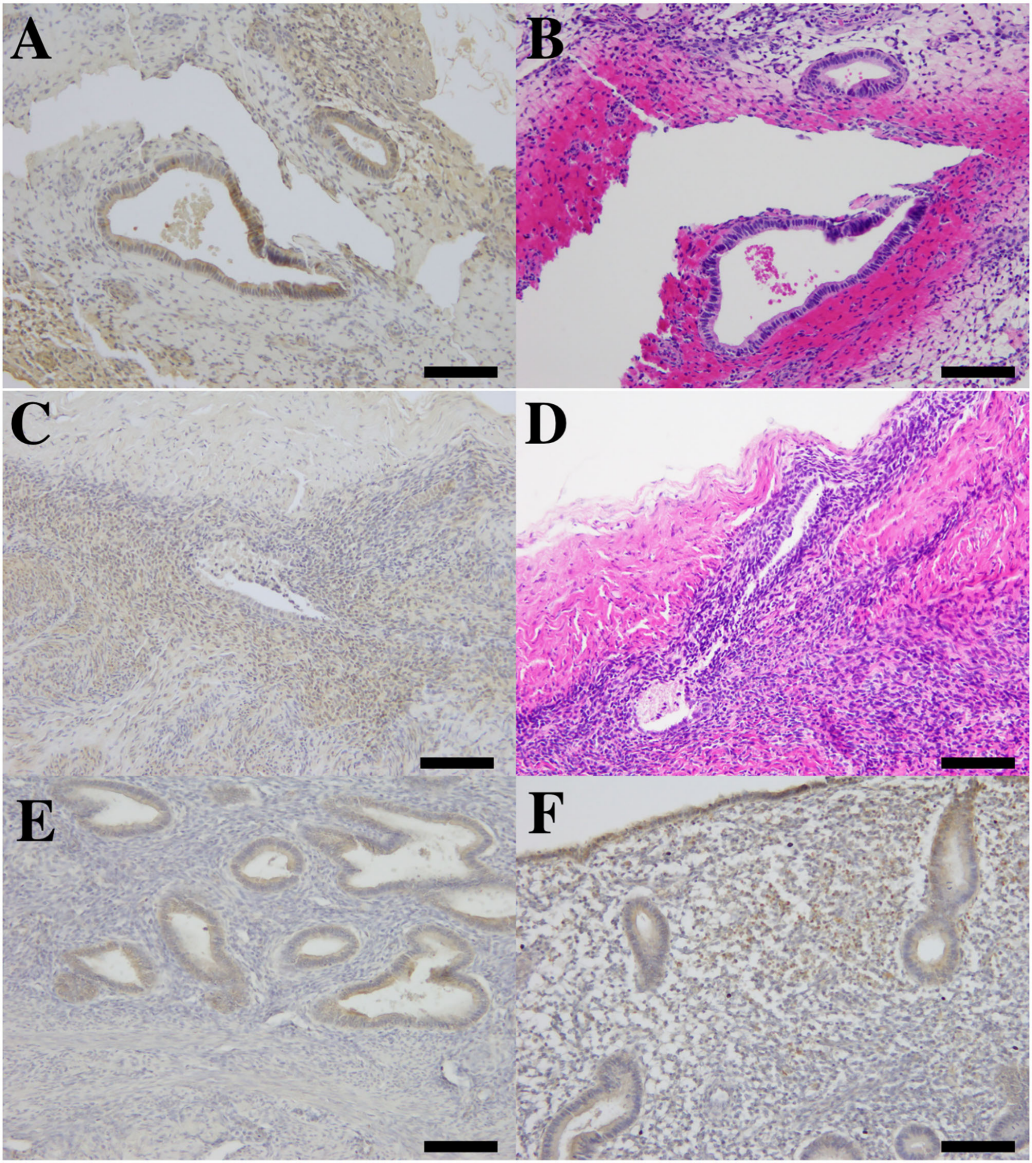
The photomicrograph of immunohistochemistry for AMHR2 and hematoxylin and eosin (H.E.) staining in ectopic and eutopic endometria. Immunolocalization of AMHR2 in human peritoneal endometriosis **(A,C)** and corresponding H.E. staining in adjacent sections **(B,D)**. Brown-colored positive immunoreactivity was found in glandular epithelium and also in endometrial stroma of peritoneal lesions **(A,C)**. We found AMHR2-positive immunoreactivity in human eutopic endometrium derived from full-thickness sampling of endometrium (positive control, **E,F**). Positive immunoreactivity was found in glandular epithelium of basalis endometrium **(E)** and in glandular epithelium and stroma of superficial functional part of eutopic endometrium **(F)**. All bars indicate 100 μm.

We found AMHR2 expression in peritoneal endometriotic lesions. The expressions were positive in glandular epithelium and stromal cells (Figures 5A–D). In the eutopic endometrium (positive control), AMHR2 are mainly localized at glandular cells and staining was more intense in the basal layer than in the functional layer (Figure 5E). AMHR2 is also strongly expressed at superficial glands and stromal cells (Figure 5F). We did not detect an immunoreaction in ectopic and eutopic endometria in negative control which replaced the primary antibody to AMHR2 to rabbit IgG (data not shown).

## Discussion

Serum AMH levels are utilized as a marker of ovarian response in the field of clinical reproductive medicine (4). On the other hand, AMH, also designated as Müllerian inhibitory substance (MIS), is originally discovered as a key molecule involved in the regression of the Müllerian duct in male fetus (3). The Müllerian duct is an embryonic precursor tissue (anlagen) of the uterus, fallopian tubes, and pelvic peritoneum. AMH may work as an inhibitor to the tissue derived from the Müllerian duct. In the study of endometrial and epithelial ovarian cancer cell lines, both may be Müllerian duct derivatives; AMH showed growth inhibitory effects on these cells (6). In addition, eutopic and ectopic endometria treated with AMH *in vitro* may decline their proliferative activity and may increase the intracellular signal of apoptosis (11–13). These previous reports may indicate that the endometrium and endometriotic tissue may be the target of AMH; however, the exact role of AMH in the growth and maintenance of endometriosis is not fully elucidated. As we show in this study, the presence of different levels of AMH in peritoneal fluids may implicate the role of these molecules in the growth and progression of peritoneal endometriosis.

Substances accumulated in peritoneal fluids may constitute secretion and permeation from the peritoneum and surrounding vascular flows. The concentrations of certain substances in peritoneal fluids may be correlated with those of serum levels. In women of reproductive age, the production from granulosa cells of ovarian follicles is solely responsible for AMH in serum because it becomes undetectable in the period of menopausal transition or after bilateral oophorectomy (14). In this present study, we found a significant correlation between serum AMH and AMH in PF in both women with endometriosis and control women without disease. AMH levels in PF and their correlation with serum AMH levels in women with and without endometriosis had been reported previously (15). Similar to our study, this study also showed a significant linear correlation between AMH in peritoneal fluids and in serum in both women with endometriosis and control women without disease. However, the severity of endometriosis was not considered in the previous report. In addition, age distributions between women with endometriosis and control women without disease significantly differed. In this study, we found significant inverse linear correlations between age and serum AMH and AMH in PF in women with endometriosis. The trends of linear correlations between age and AMH in PF in women with and without endometriosis were also reported in the previous study (15), which correspond with our present findings. AMH levels in PF may correlate with age-dependent decline in ovarian reserve.

In this study, we did not find a significant difference in serum AMH levels according to the presence of endometriosis. On the other hand, when we compare AMH in PF according to the presence of endometriosis, we found significantly lower AMH in PF in women with advanced endometriosis compared to those of control women. These results may indicate that AMH in PF may be affected by the presence of endometriosis. Although the cause of PF-specific alteration in AMH levels is unclear, lower AMH in PF in women with endometriosis may be related to pathophysiology of endometriosis. If AMH act as an inhibitor to Müllerian duct-derived tissue, lower AMH levels in PF can be interpreted as a putative growth promotive environment for disease.

In this study, we confirmed localization of a specific receptor for AMH (i.e., AMHR2) in peritoneal endometriosis. Previous study also reported the expression of AMH and AMHR2 mRNA and protein in endometrial glandular epithelium and stromal cells in endometrial tissue obtained during infertility evaluation (10). In another study, isolated endometrial stromal cells derived from ovarian endometriosis also express AMHR2, and AMH showed growth inhibition on these cells *in vitro* (11) In addition, higher AMH and AMHR2 mRNA levels in eutopic and ectopic endometria of women with endometriosis compared to those of control women without disease had been reported (16). These reports indicate that endometriotic lesion is also a target of AMH, and the effects of AMH may be more pronounced compared to those of the eutopic counterpart. Ovarian and deep infiltrated endometriosis but not peritoneal endometriotic foci were utilized in previous studies. Since these lesions may have different pathological entities (17), peritoneal endometriosis and AMH in PF may differently interact compared to other types of endometriosis. Our present study may implicate that AMH in peritoneal fluids may exert its effect via AMHR2 on peritoneal endometriosis.

In conclusion, the concentration of AMH in PF correlates with age and serum AMH levels, but these associations may be less relevant in women with endometriosis. AMH levels in peritoneal fluids may differ according to the presence of ovarian endometriosis which lowers productions of AMH from ovarian granulosa cells. Peritoneal endometriosis expresses AMHR2, the specific receptor for AMH, which may implicate a functional role of AMH in growth maintenance of these lesions.

## Data Availability Statement

The raw data supporting the conclusions of this article will be made available by the authors, without undue reservation.

## Ethics Statement

The studies involving human participants were reviewed and approved by Nagasaki University Hospital ethical committee. The patients/participants provided their written informed consent to participate in this study.

## Author Contributions

MK was an organizer of this study and the chief surgeon and was responsible for writing the manuscript. KMa was an assistant surgeon and did the sample collection and data analysis. NM worked as an outpatient manager and did data analysis. IK worked as an assistant surgeon and inpatient manager. AH worked as an assistant surgeon and inpatient manager and did the sample collection and data analysis. YK worked as an outpatient manager and data analysis. HM had roles in manuscript correction. KMi had roles in writing and correcting the manuscript. All authors contributed to the article and approved the submitted version.

## Conflict of Interest

The authors declare that the research was conducted in the absence of any commercial or financial relationships that could be construed as a potential conflict of interest.
